# Ultrasound-Mediated Membrane Modulation for Biomedical Applications

**DOI:** 10.3390/nano15120884

**Published:** 2025-06-07

**Authors:** Jinhee Yoo, Dasom Heo, Yunhee Hwang, Chulhong Kim, Byullee Park

**Affiliations:** 1 Department of Biophysics, Institute of Quantum Biophysics, Sungkyunkwan University, Suwon 16419, Republic of Korea; 2 Department of Electrical Engineering, Convergence IT Engineering, Mechanical Engineering, Chemical Engineering, Medical Science and Engineering, Graduate School of Artificial Intelligence, and Medical Device Innovation Center, Pohang University of Science and Technology, Pohang 37673, Republic of Korea

**Keywords:** ultrasound, cell membrane, cavitation, sonoporation, mechanotransduction, drug delivery, therapeutics, diagnostics

## Abstract

The cell membrane plays a critical role in regulating substance exchange, signal transduction, and energy conversion, making it essential for maintaining homeostasis and responding to environmental stimuli. Ultrasound is a non-invasive, low-toxic modality that penetrates deep tissues, offering a promising alternative to traditional physical stimuli for advancing cell membrane research. This review focuses on the approaches by which ultrasound interacts with cell membranes and highlights its diverse biomedical applications. Key approaches of ultrasound–membrane interaction include cavitation, sonoporation, and mechanotransduction, which have been harnessed in drug delivery, therapeutics, and diagnostics. Furthermore, we discuss current challenges and future directions to advance the clinical and research potential of this field. Ultrasound-mediated membrane modulation serves as a bridge between fundamental biological studies and clinical translation.

## 1. Introduction

The cell membrane is a fundamental structure that maintains the integrity and functionality of all living cells [1,2]. It is composed primarily of a phospholipid bilayer embedded with proteins and serves as a selectively permeable barrier for the transport of ions, nutrients, and signaling molecules. This dynamic exchange is essential for maintaining intracellular homeostasis and coordinating responses from external stimuli [3]. Membrane-associated receptors and ion channels mediate crucial processes such as immune activation, synaptic transmission, cell migration, and wound healing [4,5]. To modulate membrane layers for therapeutic and research purposes, a variety of techniques have been developed, including chemical treatments, genetic modifications, and physical methods [2,6,7,8]. Among these, physical interventions offer key advantages, providing spatiotemporally precise modulation without permanently altering cellular systems.

Various physical modalities have been explored for membrane modulation, including electrical stimulation, optical excitation, mechanical deformation, and magnetic manipulation [7,8]. Of these, electrical and optical techniques are most used due to their high spatial and temporal precision [9,10,11,12]. However, despite their effectiveness, these techniques face notable limitations, such as thermal or oxidative stress, potential damage to fragile biomolecules, and limited tissue penetration depth [8]. These drawbacks restrict their scalability and applicability in vivo.

Ultrasound has emerged as an alternative for membrane modulation, owing to its ability to penetrate deep tissues with minimal cytotoxicity [13,14,15], and to apply mechanical stimulation without requiring direct contact. This capability is primarily driven by acoustic pressure and microbubble dynamics, enabling transient and localized modulation of membrane integrity [13,16]. The effects of ultrasound on membrane response can be precisely and critically tuned by adjusting parameters such as frequency, intensity, and pulse duration, highlighting the importance of careful parameter optimization for effective and safe application [8,14,15]. Moreover, its demonstrated efficacy in both in vitro and in vivo settings highlights its strong potential for clinical translation.

**Figure 1:**
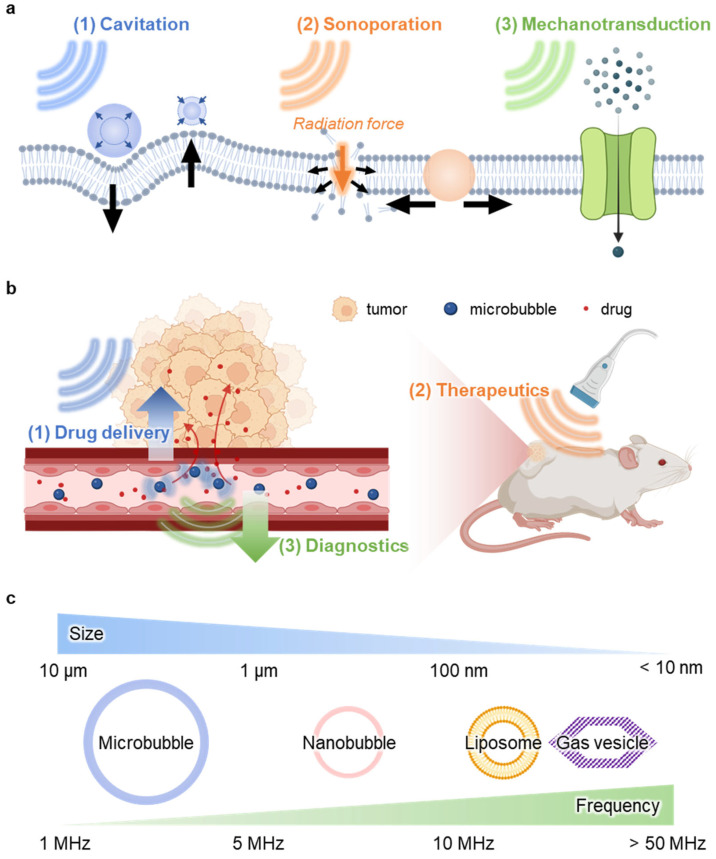
Summary of ultrasound-mediated membrane modulation. (**a**) Approaches for modulating membranes with ultrasound: cavitation, sonoporation, and mechanotransduction. (**b**) Representative biomedical applications enabled by membrane modulation: drug delivery, therapeutics, and diagnostics. (**c**) Ultrasound-responsive agents used in this study. The figure was created with BioRender.com.

This review explores the approaches and biomedical applications of ultrasound-mediated cell membrane modulation. Ultrasound induces functional changes in cellular membranes through three primary approaches: cavitation, sonoporation, and mechanotransduction (Figure 1a). Cavitation generates mechanical forces via the formation and collapse of microbubbles. Sonoporation creates transient pores that facilitate molecular transport across the membrane. Mechanotransduction converts mechanical stimuli into biochemical signals that affect cellular functions. Together, these approaches support a wide range of biomedical applications. Ultrasound enhances both the release and retention of therapeutic agents for drug delivery, modulates membrane dynamics to regulate cellular behavior in therapy, and enables sensitive and noninvasive detection in diagnostics through membrane-associated acoustic responses (Figure 1b). By linking fundamental approaches with practical applications, this review underscores the potential of ultrasound-based membrane modulation to advance the field of biomedical engineering.

As a brief reference, these applications are mediated by several types of ultrasound-responsive agents—including microbubbles, nanobubbles, liposomes, and gas vesicles (GVs)—each with distinct size scales and acoustic properties (Figure 1c) [17,18]. Microbubbles are gas-filled spheres several micrometers in size with lipid or polymer shells. Nanobubbles are smaller gas-core structures coated with lipid or surfactant coatings. Liposomes are phospholipid vesicles typically 50–200 nanometers, and gas vesicles are protein-shelled, gas-filled nanostructures that can be genetically encoded.

## 2. Approaches of Ultrasound-Mediated Membrane Modulation

### 2.1. Cavitation

**Figure 2:**
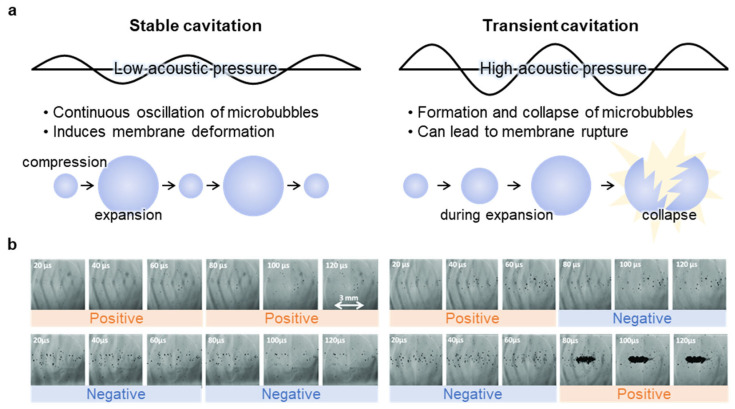
Approach of ultrasound-induced membrane modulation via cavitation. (**a**) Schematic of cavitation bubble behavior under low and high acoustic pressure conditions. Low-pressure stimulation induces stable cavitation characterized by oscillating bubbles, whereas high-pressure conditions lead to transient cavitation and bubble collapse. (**b**) High-speed images showing bubble generation and interaction under distinct pressure phase conditions. Negative pressure facilitates bubble nucleation, while subsequent positive pressure promotes clustering and amplifies cavitation activity. The combination of negative-positive pressure sequence yields the most effective cavitation enhancement. Reproduced with permission from [39].

Acoustic cavitation can be divided into stable cavitation and transient cavitation according to bubble dynamics and collapse behavior (Figure 2a) [19,20,21,22,23]. Stable cavitation occurs at low acoustic pressures, where microbubbles continuously oscillate. These oscillations are nonlinear in nature, giving rise to harmonic signals and scattering effects that are central to diagnostic ultrasound [24,25]. This process can induce reversible membrane deformation [26,27], change membrane fluidity [28], or activate mechanosensitive ion channels [29]. On the other hand, transient cavitation, which occurs at higher acoustic pressure, is accompanied by violent collapse of bubbles, generating shock waves and microjets [30], which can lead to pore formation [31] or membrane rupture. Stable cavitation provides a safe and non-destructive method to modulate membrane properties and is used for targeted drug delivery [32], blood–brain barrier (BBB) opening [33], gene delivery [34], and neuromodulation [29]. However, transient cavitation is more likely to destroy cells or membranes and is mainly used for therapeutic purposes such as targeted cell ablation [35,36], tissue dissection, and coagulation [37]. Several factors, such as the acoustic pressure, the frequency, and the temperature of the target, influence the balance between the two cavitation states. Therefore, it is important to optimize these factors to avoid unintended damage.

Acoustic pressure influences cavitation dynamics, as cavitation occurs only when a specific pressure threshold (minimum 0.3 MPa) is surpassed [38]. Cavitation behavior is significantly affected by peak positive and negative pressures. Negative pressure and positive pressure play distinct roles in bubble formation: negative pressure facilitates bubble nucleation, whereas positive pressure promotes bubble interaction and clustering, thereby amplifying cavitation effects (Figure 2b) [39]. This negative –positive pressure sequence represents the most effective approach for enhancing cavitation phenomena.

Another factor is the frequency. At low frequencies (below 100 kHz), bubbles can grow larger and are more prone to transient cavitation, generating strong shock waves and high-speed microjets [40]. In contrast, at high frequencies (above 100 kHz) produce smaller bubbles that tend to oscillate steadily without collapsing, favoring stable cavitation [41]. As a result, low-frequency ultrasound is typically used to induce strong mechanical effects, while high-frequency ultrasound enables milder and more spatially confined modulation due to its higher spatial precision.

Temperature also influences cavitation behavior by affecting the physical properties of the medium [42]. At higher temperatures, gas solubility decreases, viscosity and surface tension are reduced, and vapor pressure increases. These factors facilitate cavitation initiation but weaken bubble collapse intensity. In contrast, at lower temperatures, gas solubility increases, viscosity and surface tension rise, and vapor pressure decreases, leading to more intense bubble collapse. Therefore, high-temperature conditions are more suitable for applications requiring gentle cavitation, such as ultrasonic cleaning or drug delivery, whereas low-temperature environments are preferred for processes that benefit from strong bubble collapse, including cell disruption or sonochemical reactions. In conclusion, a comprehensive understanding of the interplay between frequency, acoustic pressure, and temperature enables the optimization of cavitation conditions for specific applications.

While temperature significantly influences cavitation dynamics, its modulation in vivo is challenging due to physiological thermoregulation, tissue heterogeneity, and limited real-time monitoring capabilities. These constraints make temperature a viable control parameter in in vitro studies, but less practical for in vivo applications where precise thermal control is often unfeasible.

### 2.2. Sonoporation

**Figure 3:**
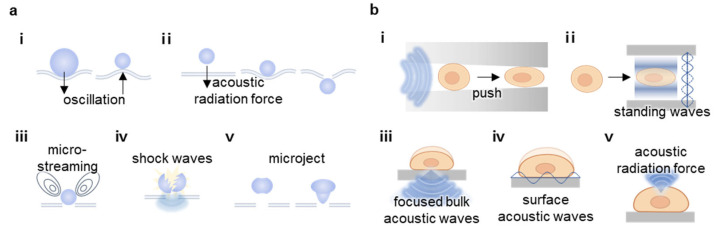
Approach of ultrasound-induced membrane modulation via sonoporation. (**a**) Bubble-mediated sonoporation: (i) Oscillating microbubbles induce membrane deformation via cavitation. (ii) Acoustic radiation force compresses the membrane. (iii) Microstreaming applies shear stress. (iv) Collapsing bubbles generate shock waves. (v) Microjets puncture the membrane. (**b**) Non-bubble-based sonoporation: (i) Traveling acoustic waves push cells through a nozzle. (ii) Standing waves create pressure gradients. (iii) Focused bulk acoustic waves induce localized deformation in adherent cells. (iv) Surface acoustic waves apply shear stress. (v) Acoustic radiation force promotes pore formation. The figure was created with BioRender.com.

Sonoporation involves using ultrasounds to create temporary nano- or micro-scale pores in the cell membrane, allowing external substances to enter [13,22,43]. In microbubble-mediated mechanisms, oscillating microbubbles generate pushing and pulling forces that deform the membrane, which is based on the cavitation process (Figure 3a(i)). Acoustic radiation force pushes microbubbles, compressing the membrane and inducing temporary disruptions (Figure 3a(ii)). Microstreaming caused by vibrating microbubbles applies shear stress, facilitating pore formation (Figure 3a(iii)). Additionally, collapsing microbubbles produce shock waves (Figure 3a(iv)) and microjets (Figure 3a(v)) that can directly penetrate the membrane.

Non-bubble-based sonoporation mechanisms offer alternative strategies to enhance cell membrane permeability without the need for microbubbles. Traveling acoustic waves can push cells through a nozzle, causing membrane deformation and pore formation (Figure 3b(i)). Standing acoustic waves create pressure fields that drive cells to regions of high acoustic pressure gradients, facilitating membrane disruption (Figure 3b(ii)). Focused bulk acoustic waves deliver concentrated energy to cells adhered to a substrate, inducing localized membrane deformation (Figure 3b(iii)). Surface acoustic waves propagate along a substrate, applying horizontal shear forces (Figure 3b(iv)). Bulk acoustic waves can also generate acoustic radiation force, promoting pore formation (Figure 3b(v)). These methods enable precise and controlled sonoporation, expanding the potential for ultrasound-based therapeutic and diagnostic applications.

Sonoporation is highly dependent on ultrasound parameters, including frequency, acoustic pressure, pulse duration, and the number of cycles. As acoustic pressure increases, the shear stress between bubbles and the cell membrane rises nonlinearly [44]. Pores form when shear stress exceeds a threshold, though higher frequencies and more cycles can induce pores under lower stress [45]. Real-time single-cell measurements have confirmed that strong sonoporation is associated with larger cavitation signals, particularly when transient cavitation occurs, which plays a key role in pore formation [46,47]. Thus, sonoporation primarily relies on cavitation, especially transient cavitation induced by strong acoustic energy, to mechanically deform the cell membrane.

An alternative method, termed acoustic transfection, has been proposed [48]. This approach utilizes high-frequency ultrasound (>150 MHz) to directly induce membrane perforation via mechanical stress without the need for microbubbles or cavitation. This technique has been shown to achieve effective sonoporation with reduced cytotoxicity. Fluorescence imaging confirms the efficacy of sonoporation by demonstrating the intracellular delivery of fluorescent markers. Therefore, bubble-free sonoporation is gaining attention as a precise, noise-free, and cell-friendly approach for intracellular delivery.

### 2.3. Mechanotransduction

**Figure 4:**
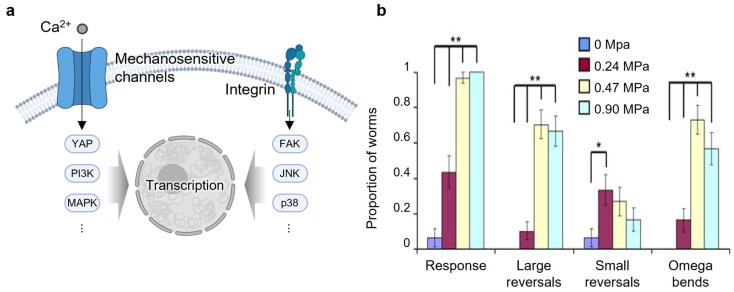
Approach of ultrasound-induced membrane modulation via mechanotransduction. (**a**) Schematic illustration of two primary ultrasound-responsive mechanotransduction routes: mechanosensitive ion channels and integrin-mediated signaling. The figure was created with BioRender.com. (**b**) TRP-4-expressing neurons in *C. elegans* exhibit distinct activation patterns under varying acoustic pressure. * *p* <0.05 and ** *p* <0.01. Reproduced with permission from [49].

Ultrasound-induced membrane deformation can initiate intracellular signaling through two primary mechanotransduction pathways: activation of mechanosensitive ion channels and integrin-mediated signaling (Figure 4a). In the channel-mediated route, ultrasound physically stimulates the lipid bilayer, directly gating mechanosensitive channels such as TRP, Piezo, and MscL [49,50,51,52,53,54]. These channels convert mechanical forces into biochemical signals by mediating calcium influx or ATP release, subsequently triggering intracellular cascades including IP_3_-mediated calcium release or calcium-dependent transcriptional regulation.

In the second mechanism, membrane-bound integrins sense mechanical stress and transduce it into the cytoskeleton. Upon ultrasound stimulation, integrins initiate intracellular signaling by recruiting focal adhesion components, such as focal adhesion kinase (FAK), which mediate the activation of downstream cascades. In synovial cells, low-intensity pulsed ultrasound activates β1 integrins, leading to a FAK–MAPK signaling cascade involving ERK, JNK, and p38 [55]. These integrin-mediated pathways convert membrane-level mechanical signals into biochemical responses for tissue repair and regeneration.

Similar to cavitation and sonoporation, the effectiveness of ultrasound-induced mechanotransduction depends strongly on acoustic parameters such as frequency, intensity, and duration. In TRP- and MscL-expressing neurons, activation thresholds and response profiles vary with ultrasound settings, with specific frequencies and pressures producing optimal effects [49,52]. For example, TRP-4-mediated neuronal activation in *C. elegans* exhibits distinct response profiles depending on the applied acoustic pressure (Figure 4b). In other studies, increased ultrasound intensity enhances calcium release through PANX1 channels and stimulates ATP release via connexin 43 hemichannels, leading to calcium wave propagation [53,54]. In integrin-mediated pathways, both intensity and exposure time significantly influence downstream signaling. Low-intensity pulsed ultrasound activates integrin-associated cascades in synovial and osteoarthritic chondrocytes, including FAK–MAPK and FAK–PI3K–Akt pathways, with longer exposures enhancing ECM-related responses, as evidenced by increased p-FAK levels [55,56].

## 3. Biomedical Applications of Ultrasound-Mediated Membrane Modulation

### 3.1. Drug Delivery

Ultrasound can serve as an effective modality to improve drug delivery efficiency and target specificity. It can alter the physical properties of drug carriers or cell membranes, thereby facilitating tissue penetration, intracellular uptake, and the overcoming of biological barriers. These techniques are commonly applied across various drug delivery applications, such as tumor therapy, transient opening of the BBB, and gene delivery, each of which requires an optimized approach depending on the specific physiological context (Table 1).

#### 3.1.1. Tumor Treatment

Ultrasound-assisted drug delivery enhances tumor therapeutic efficacy by combining the effects of cavitation and sonoporation [66,67]. Cavitation, particularly in the presence of microbubbles, promotes drug release from carriers such as micelles or liposomes through mechanical disruption. Concurrently, sonoporation increases cell membrane permeability, facilitating intracellular drug accumulation. This dual approach allows for efficient drug transport into tumor tissues and improves distribution within the tumor microenvironment. This approach offers several advantages over other delivery methods, including noninvasive administration, spatial and temporal targeting, and enhanced intracellular uptake [68,69,70,71].

**Figure 5:**
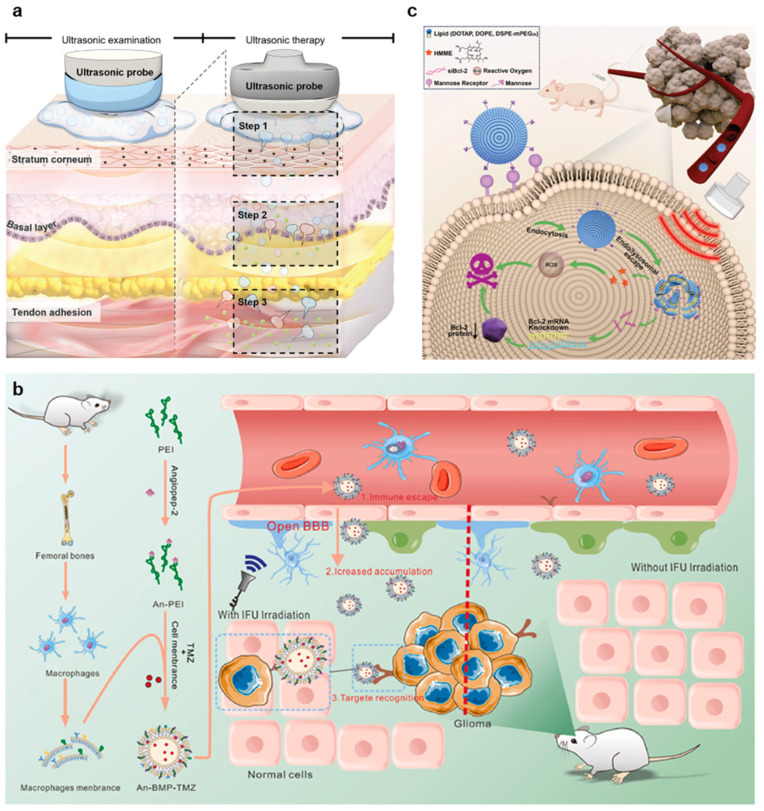
Application of membrane modulation for drug delivery. (**a**) Deep-tissue drug delivery enabled by nanobubble–liposome coupling agents. Upon ultrasound stimulation, nanobubbles permeabilize cell membranes and facilitate the release of therapeutic agents to otherwise inaccessible layers without invasive procedures. (**b**) Ultrasound-enhanced BBB opening for glioma-targeted delivery. Macrophage membrane-coated nanoparticles cross the BBB under ultrasound irradiation, enabling immune evasion, increased accumulation, and selective delivery to glioma tissues. (**c**) Ultrasound-sensitive liposomal system for gene therapy. Targeted liposomes respond to ultrasound by enhancing membrane permeability and facilitating intracellular delivery of genetic material, enabling mRNA knockdown and improved tumor treatment efficacy. Reproduced with permission from [57,76,80].

Recent advances in ultrasound-mediated tumor therapy have underscored the membrane as a dynamic interface that can be modulated to enhance drug delivery. For instance, nanobubble–liposome coupling agents have enabled deep-tissue delivery by facilitating ultrasound-triggered membrane permeabilization (Figure 5a) [57]. Similarly, ultrasound-responsive nanogels have been developed to modulate membrane permeability and release chemotherapeutic payloads directly into tumor cells, while concurrently reprogramming the tumor microenvironment to improve treatment outcomes [58]. In orthotopic liver tumors, nanobubbles have shown greater membrane interaction and intracellular drug accumulation than microbubbles, suggesting that nanoscale carriers can more effectively interface with tumor membranes under ultrasound stimulation [59]. Together, these studies reflect a shift toward designing ultrasound-responsive systems that actively engage with the membrane to achieve spatially controlled, efficient therapeutic delivery.

#### 3.1.2. Blood–Brain Barrier Opening

The BBB presents a major challenge to drug delivery for central nervous system disorders [72]. Composed of tightly connected endothelial cells, the BBB strictly regulates molecular transport between the bloodstream and brain parenchyma, effectively protecting neural tissue from toxins and pathogens. FUS, especially when combined with microbubbles, offers a noninvasive approach to temporarily and locally open the BBB by inducing mechanical stress on the endothelial membrane [68]. This stress disrupts tight junctions, increasing paracellular permeability, and may also modulate transcellular transport by enhancing vesicular trafficking and membrane transporter activity [72,73,74]. While clinical studies have confirmed the feasibility and safety of this technique in neurodegenerative diseases, achieving precise spatiotemporal control is essential to ensure consistent BBB opening and to minimize risks such as neuroinflammation or vascular injury [75].

Recent trends in ultrasound-mediated BBB opening have increasingly focused on membrane-level modulation strategies to achieve more precise and efficient control of permeability. Macrophage membrane-coated nanoparticles enable targeted delivery by leveraging membrane affinity and ultrasound responsiveness to facilitate glioma-specific drug release (Figure 5b) [76]. Another study utilizes ultrasound-activated microbombs that bind directly to endothelial surfaces and disrupt the BBB upon stimulation, minimizing off-target effects [60]. Meanwhile, piezoelectric sonosensitizers have demonstrated precise mitochondrial membrane depolarization in glioma cells under ultrasound, highlighting intracellular membrane targets as therapeutic interfaces [61]. Complementing these innovations, molecular simulations of shockwave-induced membrane deformation have provided insight into nanoscale mechanical responses of lipid bilayers, guiding rational device design [62].

#### 3.1.3. Gene Delivery

Ultrasound-mediated gene delivery leverages membrane perturbation to enhance the intracellular uptake of nucleic acids. This approach uses acoustic stimulation to transiently alter membrane permeability, enabling efficient delivery of DNA, RNA, or siRNA to target cells [34,77,78,79]. The cell membrane serves as a controllable interface whose mechanical and biophysical properties can be modulated by ultrasound to facilitate gene transfer. This strategy offers advantages in safety, spatial control, and repeatability, while avoiding immune activation [79].

Recent advances in ultrasound-mediated gene delivery have focused on modulating the cell membrane to enhance gene transfer. For instance, ultrasound-sensitive liposomes and targeted viral vectors leverage ultrasound to transiently open cell membranes, facilitating the efficient delivery of therapeutic genes to liver tumors and hemophilia A treatment (Figure 5c) [63,65,80]. Moreover, ultrasound-responsive nanobubbles are employed for siRNA delivery to bone tissues, enabling effective gene-based therapies in osteoporosis [64]. These studies highlight the growing potential of ultrasound in modulating cell membranes, ensuring precise, non-invasive, and controlled gene delivery for various therapeutic applications.

### 3.2. Therapeutics

Ultrasound can also be used as a therapeutic tool by modulating cell signaling and tissue responses. It primarily acts through mechanotransduction to promote the expression of specific genes or to regulate secondary messengers. These effects support a range of therapeutic applications, including neuromodulation, immunotherapy, and tissue regeneration (Table 2).

#### 3.2.1. Neuromodulation

Ultrasound-mediated nerve stimulation modulates calcium influx, enabling precise and reversible control of neural activity [95]. A key development in this field is sonogenetics, which combines ultrasound with genetically introduced ultrasound-sensitive proteins to selectively activate targeted neuronal populations [96,97]. Engineered mechanosensitive channels in the membrane convert acoustic forces into electrophysiological responses with improved sensitivity, precision, and reduced off-target effects [98,99]. This approach offers noninvasive, cell-specific neuromodulation with high spatial resolution, making it a promising alternative to conventional electrical stimulation [100].

**Figure 6:**
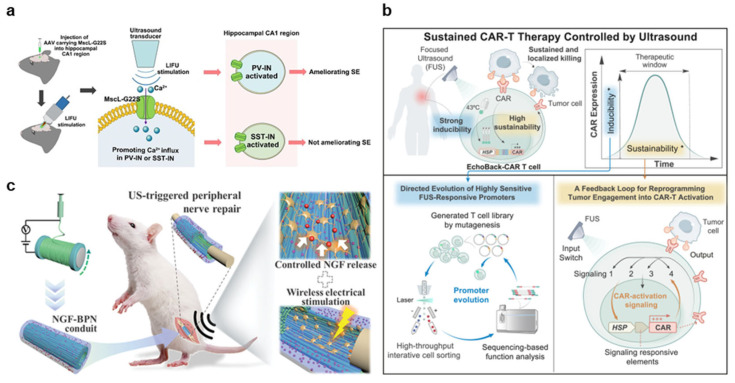
Application of membrane modulation for therapeutics. (**a**) Sonogenetic neuromodulation via membrane-targeted ion channels. Ultrasound activates mechanosensitive channels (e.g., Mscl-G22S) in specific neurons, inducing calcium influx and enabling selective, noninvasive control of neural activity. (**b**) Ultrasound-controlled CAR-T cell therapy. Focused ultrasound induces gene expression through thermal-sensitive promoters in engineered T cells, enabling programmable, localized activation of anti-tumor immunity. (**c**) Ultrasound-triggered peripheral nerve repair using an NGF conduit that releases growth factors and generates electrical cues. It promotes axonal regeneration in vivo. Reproduced with permission from [83,85,88].

Recent advancements in sonogenetics have focused on engineering membrane-bound mechanosensitive channels and optimizing acoustic targeting strategies to achieve cell-specific, safe, and spatially precise neuromodulation. A notable trend is the engineering of mechanosensitive ion channels that can be expressed in specific neural populations, such as TRPV1 and GABAergic neurons, to lower the acoustic threshold for activation and reduce unintended stimulation of surrounding tissue (Figure 6a) [81,82,83]. For instance, the activation of TRPV1 channels in the motor cortex has demonstrated precise control of locomotor behavior, showcasing improved spatial resolution through membrane-tethered protein gating [81]. Simultaneously, studies employing sonogenetic control of GABAergic interneurons in epilepsy models have shown that membrane-specific ultrasound activation can restore inhibitory tone and suppress seizure activity [82,83]. To address the challenge of targeting neural populations with greater spatiotemporal flexibility, recent work has incorporated Airy-beam holography into sonogenetics, enhancing mechanotransduction by shaping pressure fields in three dimensions [84].

#### 3.2.2. Immunotherapy

Ultrasound-mediated immunotherapy leverages mechanical forces generated by acoustic waves to modulate immune cell behavior. Ultrasound-mediated modulation of membrane permeability and mechanosensitive channels enables nano-vaccine delivery and STING pathway activation, enhancing immune responses [101,102]. This approach enables spatially and temporally controlled stimulation of immune responses without the need for invasive procedures or systemic toxicity, making it a promising strategy for tumor immunotherapy [103,104]. Additionally, ultrasound can induce immunogenic cell death, triggering the release of tumor-associated antigens and promoting antigen presentation by dendritic cells [17]. Despite these advantages, challenges remain in achieving cell-type specificity and avoiding off-target inflammatory responses, which require careful modulation of delivery strategies [101,104].

Ultrasound-mediated immunotherapy is evolving from passive membrane disruption to active immune programming through engineered gene circuits and membrane-disruptive cell death mechanisms. At the cellular level, engineered immune cells have been designed to express synthetic heat-sensitive gene circuits [85]. This system enables remote and programmable control of CAR expression through thermal-responsive promoters, allowing spatially confined, on-demand immunomodulation. Some platforms induce immunogenic forms of cell death, such as pyroptosis, which involve catastrophic membrane rupture and subsequent antigen release, further promoting dendritic cell activation and tumor-specific immune priming [86,87].

#### 3.2.3. Regeneration

Ultrasound modulates membrane tension, activating mechanosensitive pathways such as MAPK and PI3K/Akt, which govern proliferation, migration, and differentiation—core components of tissue repair [105,106]. In skeletal muscle, LIPUS enhances regeneration by downregulating the inflammatory microenvironment and reducing immune-mediated damage [107,108]. Similarly, LIPUS facilitates bone healing through osteoblast stimulation and modulation of the OPG/RANKL [109,110]. In peripheral nerve injuries, ultrasound promotes Schwann cell activity and axonal regrowth, accelerating reinnervation in autograft models [111,112,113]. Additionally, ultrasound-targeted microbubble destruction enables site-specific gene delivery, supporting regenerative outcomes such as β-cell recovery and glucose regulation [114,115]. These modalities offer noninvasive, spatiotemporally controlled activation of endogenous repair mechanisms.

Recent advancements in ultrasound-mediated regeneration highlight the cell membrane as an active transducer of therapeutic mechanical and electrical signals. Studies have shown that piezoelectric and mechanoresponsive materials integrated with ultrasound can locally manipulate membrane potential and curvature to modulate mechanosensitive signaling cascades. For example, ultrasound-responsive piezoelectric nanofibers and hydrogels have been designed to generate localized electrical fields upon stimulation, effectively restoring physiological microenvironments and promoting tissue-specific regeneration (Figure 6c) [88,89,90]. These materials influence channel gating, leading to improved proliferation and differentiation. Additionally, several recent strategies employ nanostructures that respond to ultrasound, shifting macrophage polarization and enhancing osteoimmune conditions for bone repair [91,92]. Notably, these interventions have extended to irregular and aged tissues, where ultrasound-induced membrane signaling can compensate for impaired immune or mechanical responsiveness [93,94].

### 3.3. Diagnostics

Ultrasound-based diagnostic techniques can be enhanced by exploiting membrane-related interactions to improve contrast, specificity, and molecular sensitivity. Representative strategies include targeted microbubbles, GVs, and passive cavitation imaging (PCI), each of which offers unique advantages in detecting membrane-associated features and characterizing the cellular microenvironment *(*Table 3*)*. These processes primarily rely on cavitation phenomena, where acoustic interactions with microbubbles or GVs generate detectable signals through stable or transient oscillations. By amplifying local acoustic responses, they enable high-resolution, real-time imaging.

#### 3.3.1. Targeted Microbubble

Targeted microbubbles are gas-filled contrast agents functionalized with ligands that bind specifically to membrane-associated biomarkers, enabling ultrasound molecular imaging with high spatial specificity [130,131]. Systemically administered microbubbles circulate and attach to membranes overexpressing target molecules, such as integrins or growth factor receptors, in diseases like cancer or thrombosis [131,132,133]. Their binding is governed by ligand–receptor interactions at the membrane surface, allowing localization of disease-specific signatures without requiring extravasation [132]. In a diagnostic context, targeted microbubbles enable the detection of molecular patterns, reflecting disease presence, progression, or response to therapy [134,135]. These imaging signals are generated through stable cavitation, where microbubbles oscillate in response to ultrasound pressure, producing nonlinear acoustic scattering that enhances contrast. This approach offers several advantages: it is noninvasive, real-time, and repeatable, with minimal systemic toxicity [130,131,136]. However, the short circulation of half-life of microbubbles, limited depth penetration in some tissues, and sensitivity to hemodynamic shear forces can reduce targeting efficiency [136,137]. Additionally, membrane accessibility and antigen density on the target tissue are critical factors influencing signal fidelity and reproducibility [135,138]. Despite these challenges, targeted microbubbles represent a powerful platform for interrogating the cell membrane in vivo, offering a functional window into membrane-associated pathology with clinical translational potential.

**Figure 7:**
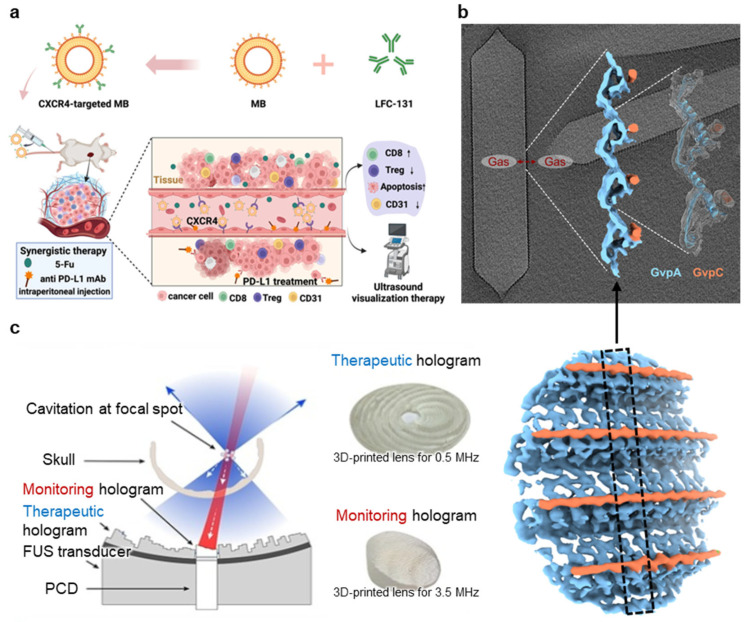
Application of membrane modulation for diagnostics. (**a**) Ultrasound imaging with CXCR4-targeted microbubbles. Ligand-functionalized microbubbles bind to CXCR4-expressing tumor vasculature, enabling noninvasive ultrasound molecular imaging and guiding immunochemotherapy. (**b**) Protein-based GVs for nanoscale ultrasound imaging. Genetically encoded GVs with membrane-like protein shells (GvpA/GvpC) exhibit acoustic contrast and structural stability for multiplexed imaging applications. (**c**) PCI with 3D-printed acoustic holograms. Therapeutic and monitoring holograms enable simultaneous ultrasound treatment and cavitation monitoring, improving precision in therapies. Reproduced with permission from [118,120,129].

Recent advances have extended the functional capacity of targeted microbubbles by engineering them to probe distinct features beyond simple ligand–receptor binding. For instance, microbubbles tuned to different resonance frequencies have been shown to selectively bind to multiple membrane biomarkers within a single imaging session, enabling multicolor ultrasound molecular imaging of both P-selectin and integrin αvβ3 on endothelial surfaces [116]. This frequency-selective targeting approach enhances molecular resolution while maintaining spatial specificity. In another application, ultrasound molecular imaging with B7-H3-targeted microbubbles was employed to guide and enhance high-intensity focused ultrasound ablation in liver cancer (Figure 7a) [117]. Similarly, CXCR4-targeted microbubbles have been developed for ultrasound imaging of membrane receptors linked to tumor aggressiveness, enabling noninvasive assessment of therapeutic response [118]. In a separate study, CD93-targeted microbubbles enabled ultrasound imaging of immunosuppressive tumor vasculature, with signal intensity reflecting the immune microenvironment and supporting the value of membrane-associated markers for diagnosis [119]. Collectively, these studies illustrate a shift from single-target, static imaging to dynamic, multi-marker membrane interrogation, positioning the cell membrane as both a diagnostic interface and a functional indicator of disease state.

#### 3.3.2. Gas Vesicle (GV)

GVs are genetically encodable, gas-filled protein nanostructures derived from aquatic microorganisms, exhibiting unique acoustic properties that enable their use as nanoscale ultrasound contrast agents [18,139]. The GV membrane, composed of a rigid, lipid-free protein shell, provides structural integrity and enables efficient ultrasound scattering, similar to that observed in microbubbles [140]. It enables advanced imaging strategies such as burst-mode detection and spectral unmixing, which enhance sensitivity and support multiplexed visualization of different cell types [141,142,143]. However, limitations such as relatively low acoustic impedance contrast and susceptibility to immune clearance remain [144].

Recent advances in GV-based ultrasound imaging have increasingly focused on engineering the GV membrane to enhance biological interactions, targeting specificity, and in vivo stability. Detailed structural studies using cryo-electron tomography have revealed that Anabaena flos-aquae GVs are composed of long, cone-tipped cylinders with highly ordered protein shells, highlighting how membrane architecture governs mechanical stability and acoustic responsiveness (Figure 7b) [120]. In addition, GV membranes have been functionalized with tumor-associated enzyme-responsive elements, enabling environment-specific signal amplification in pathological tissues [121]. Building on these structural insights, recent work has transformed GVs from passive scatterers into bioengineered imaging agents that actively interact with cellular membranes and biological barriers. For instance, PEI-coated GVs functionalized with fluorescent dyes demonstrated improved BBB penetration and dual-modal imaging performance in glioma models, showing how surface modifications enhance tissue accessibility [122]. Similarly, genetically miniaturized GVs (~50 nm) enabled lymphatic cell access, underscoring the critical role of membrane mechanics in size-dependent imaging performance [123].

#### 3.3.3. Passive Cavitation Imaging (PCI)

PCI is a passive ultrasound technique that detects acoustic emissions from microbubbles undergoing cavitation during therapeutic ultrasound exposure [145,146]. Using array-based receivers and beamforming, PCI localizes cavitation activity, making it well-suited for monitoring therapies such as histotripsy and HIFU. A key application of PCI is in evaluating ultrasound–membrane interactions, where cavitation can transiently disrupt cell membranes, enabling targeted drug or gene delivery [147,148]. However, it remains limited by axial resolution and sensitivity to acoustic heterogeneity [146,149]. Despite these challenges, PCI offers a valuable tool for guiding and understanding membrane-level bioeffects of ultrasound [147,150].

Recent advances in PCI have significantly improved its ability to monitor ultrasound–membrane interactions with greater precision and clinical relevance. To overcome limitations in axial resolution and contrast, novel beamforming techniques such as minimum variance–based methods, cross-spectral matrix inversion, and p-th root compression algorithms have been introduced, enabling more accurate spatial localization of cavitation [124,125,126]. High-resolution 2D and 3D PCI systems now offer real-time mapping of cavitation dynamics in tissue-mimicking and in vivo models, revealing how cavitation intensity and distribution correlate with membrane permeabilization [125,127,128]. Super-resolution PCI improves spatial localization of cavitation activity to tens of microns, allowing visualization of bubble dynamics near cellular interfaces and offering indirect insights into ultrasound-induced membrane effects [128]. Clinically adaptable platforms that integrate PCI with conventional B-mode imaging also support monitoring in complex settings such as thrombolysis or BBB opening (Figure 7c) [125,129]. Therefore, these innovations position PCI as a powerful tool for visualizing and guiding ultrasound-induced bioeffects.

## 4. Challenges and Future Perspectives

Ultrasound-mediated membrane modulation holds promise for drug delivery and therapeutic applications due to its non-invasive nature. However, this same non-invasiveness often limits real-time observation of its effects, especially in vivo. In animal models, outcomes are typically evaluated post-treatment, which hinders dynamic monitoring during intervention. This challenge can be overcome by integrating drug delivery and therapeutic strategies with diagnostic modalities, as discussed in Section 3.3. For instance, functionalized microbubbles can enable real-time monitoring by altering their ultrasound signal in response to drug release or therapeutic changes. Such integration highlights the potential of ultrasound as a theranostic platform for membrane-targeted biomedical interventions.

Moreover, even when ultrasound does not directly interact with the membrane, it can still provide valuable tools for membrane-related studies. Acoustic tweezers, while traditionally used for manipulating entire cells or microparticles, have shown the potential to mechanically interact with membrane-associated structures [151,152]. Studies have even demonstrated their ability to induce microbubble collapse, suggesting that they may be adapted for precise, contactless membrane manipulation [153]. On the other hand, photoacoustic imaging, a hybrid modality that converts absorbed optical energy into ultrasound signals, can provide functional information about membranes using targeted nanoparticles [154,155,156,157,158,159]. When functionalized to mimic or bind specific membrane components, these particles enable real-time, high-resolution imaging of membrane-related dynamics such as receptor expression, ion flux, or changes in membrane potential [154,160,161,162]. These capabilities have been applied to various models, including small animals and human skin tissues, with recent advances in spectroscopic and molecular imaging further enhancing targeting precision and functional resolution [163,164,165,166,167,168,169,170,171]. These technologies extend the utility of ultrasound beyond modulation, offering complementary platforms for probing membrane structure, function, and biomechanics [172,173].

We also anticipate that ultrasound-based membrane modulation research will gain broader applicability in biomedical engineering. Most membrane modulation studies are currently conducted in vitro, largely due to the subcellular scale of membranes and the high spatial precision required for analysis. However, the studies reviewed in this paper include not only in vitro experiments but also animal model applications. This broader application is likely due to the non-invasive and user-friendly nature of ultrasound compared to other membrane manipulation techniques. Furthermore, since ultrasound is already widely used in clinical practice, it holds considerable potential for translational research. We expect that ultrasound will serve as a bridge to expand membrane modulation studies toward clinical applications.

## 5. Limitations of Ultrasound-Mediated Membrane Modulation

Ultrasound-based membrane modulation offers significant advantages for biomedical applications. However, several technical and biological limitations must be addressed to ensure its broader and safer clinical use. With continued innovation, optimization, and systematic validation, these challenges can be overcome to enable effective clinical translation.

A key technical limitation is the relatively low spatial resolution of ultrasound compared to optical techniques. Although membrane structures are sub-wavelength and do not inherently require high resolution, precise and specific modulation within targeted tissues remains challenging. This may be addressed by developing advanced technologies such as high-frequency stimulation systems or beamforming strategies optimized for high-resolution control [128,153,174]. In addition, accurately focusing ultrasound energy in deep tissue is difficult due to tissue heterogeneity, acoustic attenuation, and refraction, which reduce energy delivery and targeting precision. These issues collectively increase the risk of unintended effects on surrounding healthy tissues, especially when high-intensity ultrasound is used. Notably, cavitation-induced stress can disrupt cells, damaging red blood cells and vascular endothelium [175,176]. The use of microbubbles, especially as contrast agents, further lowers the cavitation threshold and increases the risk of hemolysis and local tissue injury. To ensure both safety and efficacy, stimulation parameters such as intensity, duty cycle, and pulse mode must be carefully optimized [97,177]. Real-time monitoring of acoustic pressure, thermal effects, and cavitation activity is crucial to prevent unintended tissue damage yet remains technically limited in many in vivo settings [178,179,180].

From a biological perspective, variability in in vivo responses presents another major challenge. While ultrasound modulation is versatile—affecting immune activity, intracellular signaling, and tissue death—these effects often interact in complex and context-dependent ways. Physiological factors such as tissue composition, blood flow, thermoregulation, and immune status can lead to inconsistent outcomes. Standardized animal models and personalized stimulation protocols may improve reproducibility. Moreover, the long-term effects of repeated or chronic ultrasound exposure remain poorly understood. Longitudinal in vivo studies are needed to assess cumulative impacts on cellular function and tissue integrity, and to establish safe exposure limits for clinical applications [181].

## 6. Conclusions

Ultrasound modulates cell membranes through mechanical approaches such as cavitation, sonoporation, and mechanotransduction, enabling non-invasive and precise control. Building on these approaches, this review has discussed three major biomedical applications including drug delivery, therapeutics, and diagnostics. The ability of ultrasound to both modulate and monitor membrane dynamics highlights its potential as a theranostic tool. With its clinical accessibility and scalability, ultrasound offers a promis-ing pathway from fundamental membrane research to translational biomedical engineering.

## Figures and Tables

**Table 1:** Recent advances in drug delivery applications.

Application	Strength	Approach	Reference
Tumor treatment	- Deep tissue penetration - Spatially controlled drug release - Minimal systemic toxicity	Cavitation and sonoporation	Han et al., 2024 [57] Cui et al., 2024 [58] Nittayacharn et al., 2024 [59]
BBB opening	- Reversible and localized barrier disruption - Noninvasive - Suitable for CNS	Cavitation and sonoporation	Li et al., 2024 [60] Huang et al., 2024 [61] Zhou et al., 2024 [62]
Gene delivery	- Safe - Repeatable transfection - Low immunogenicity	Sonoporation	Lawton et al., 2024 [63] Sotoudehbagha et al., 2025 [64] Li et al., 2024 [65]

**Table 2:** Recent advances in therapeutic applications.

Application	Strength	Approach	Reference
Neuromodulation	- Cell-type specificity - Spatiotemporally precise targeting - Noninvasive	Mechanotransduction	Xu et al., 2023 [81] Phan et al., 2025 [82] Xu et al., 2024 [83] Hu et al., 2024 [84]
Immunotherapy	- Deep penetration - Localized activation - Programmable immune control	Mechanotransduction	Liu et al., 2025 [85]
		Cavitation	Sun et al., 2024 [86] Wang et al., 2024 [87]
Regeneration	- Endogenous pathway-mediated repair promotion - Sustained bioelectric signaling - Inflammation modulation	Mechanotransduction	Xu et al., 2024 [88] Ricotti et al., 2024 [89] Wang et al., 2024 [90] Zhang et al., 2024 [91] Jiang et al., 2024 [92] Zhou et al., 2024 [93] Dunham et al., 2024 [94]

**Table 3:** Recent advances in diagnostic applications.

Application	Strength	Approach	Reference
Targeted microbubble	- Targeted molecular imaging - Immune sensitization	Cavitation (stable)	Castillo et al., 2025 [116] Xiong et al., 2024 [117] Qiu et al., 2025 [118] Wang et al., 2024 [119]
Gas vesicle	- Genetically encodable - Acoustic reporter for deep tissue	Cavitation (stable)	Dutka et al., 2023 [120] Garrute et al., 2025 [121] Li et al., 2025 [122] Shen et al., 2024 [123]
Passive cavitation imaging	- Real-time mapping - Quantitative monitoring - Clinical integration	Cavitation (stable)	Singh et al., 2025 [124] Zhang et al., 2024 [125] Lachambre et al., 2024 [126] Zhu et al., 2024 [127] Li et al., 2024 [128] Lamothe et al., 2024 [129]
